# Inhalation of Ultrafine Zinc Particles Impaired Cardiovascular Functions in Hypertension-Induced Heart Failure Rats With Preserved Ejection Fraction

**DOI:** 10.3389/fbioe.2020.00013

**Published:** 2020-01-24

**Authors:** Fangbo Bing, Xuan Wang, Wenzeng Shen, Li Li, Pei Niu, Ying Chen, Wenxi Zhang, Wenchang Tan, Yunlong Huo

**Affiliations:** ^1^Department of Mechanics and Engineering Science, College of Engineering, Peking University, Beijing, China; ^2^College of Medicine, Hebei University, Baoding, China; ^3^Shenzhen Graduate School, Peking University, Shenzhen, China; ^4^PKU-HKUST Shenzhen-Hong Kong Institution, Shenzhen, China; ^5^Institute of Mechanobiology and Medical Engineering, School of Life Sciences and Biotechnology, Shanghai Jiao Tong University, Shanghai, China

**Keywords:** HFpEF, PWV, WSS, Windkessel model, Womersley analysis

## Abstract

Although it is possible for inhalation of ultrafine particles to impair human health, its effect is not clear in patients with HFpEF. This study investigated cardiac and hemodynamic changes in hypertension-induced rats of HFpEF after inhaling ultrafine zinc particles for a while. Multiple experimental measurements were carried out in DSS rats fed with high salt (HS) and low salt (LS) diets as well as HS diet with the inhalation of ultrafine zinc particles (defined as HP). Cardiac strain and strain rate were quantified by the speckle tracking echocardiography. The pressure and flow waves were recorded in the carotid artery and abdominal aorta and analyzed by the models of Windkessel and Womersley types. HS and HP rats were found to show lower strains on endocardium and epicardium than LS rats. The inhalation of ultrafine zinc particles further reduced the strain in the longitudinal direction on the endocardium of rats with HFpEF, but had relatively small effects on the epicardium. The inhalation of ultrafine zinc particles resulted in the increase of systemic resistance and the decrease of total vascular compliance as well as the increased PWV and induced more severe vascular stiffening in rats with HFpEF. In summary, the inhalation of ultrafine zinc particles deteriorated local myocardial dysfunctions in the LV and the hemodynamic environment in peripheral arteries in rats of HFpEF. This study is of importance to understand the mechanisms of cardiovascular impairments owing to air pollution.

## Introduction

Air quality has recently become a major concern in China. The exposure to particulate matter air pollution, particularly in PM0.1 (ultrafine particles), significantly deteriorates cardiovascular diseases and heart diseases (Mills et al., 2009; Shah et al., 2013; Hwang et al., 2014; Brook et al., 2018). The ultrafine particles contain a large amount of metal components, which can be a key risky factor for deterioration of the cardiovascular diseases (Birmili et al., 2006; Kodavanti et al., 2008; Wallenborn et al., 2008). The morbidity and mortality of heart failure (HF) with preserved ejection fraction (HFpEF) are rising (Sharma and Kass, 2014). There is, however, lacking of studies to investigate the effects of PM0.1 on patients with HFpEF.

The dahl salt-sensitive (DSS) rat is hypersensitive to sodium intake and a good experimental model for the study of hypertension-induced HFpEF (Klotz et al., 2006; Gomes et al., 2013). When placed with high salt (HS) diet for 7 weeks, the HS-feeding DSS rat has shown the elevated diastolic LV stiffness and slow LV relaxation, two important factors for the diagnosis of diastolic LV dysfunction by both the European and American Echocardiography Associations (Paulus et al., 2007; Nagueh et al., 2016), as well as the impaired ventricular–vascular coupling, vascular dysfunction, and reduction of active contracting stress (Yin et al., 2019). Inhaling metal components, e.g., ultrafine zinc particles, leads to vascular dysfunctions by increasing endothelial oxidative stress (Mills et al., 2005; Wauters et al., 2013) such that it may accelerate the development of HFpEF. As a logic starting point, HS-feeding DSS rats were made inhaling ultrafine zinc particles to find the hemodynamic mechanisms for potential deterioration of HFpEF due to air pollution.

The objective of the study is to investigate cardiovascular changes of HS-feeding DSS rats after inhaling ultrafine zinc particles for 4 weeks. Here, we hypothesize that inhalation of ultrafine zinc particles can worsen LV dysfunction and hemodynamics in peripheral arteries in rats with HFpEF. To test this hypothesis, the DSS rats were placed on HS diet for 7 weeks and inhaled ultrafine zinc particles for 4 weeks. Physiological and hemodynamic measurements were carried out in the LV, abdominal aorta and carotid artery. Speckle tracking echocardiography (STE) was used to analyze regional and global ventricular functions (Voigt et al., 2015). The Windkessel and Womersley models were performed for the hemodynamic analysis in the abdominal aorta and carotid artery (Zheng et al., 2010; Huo et al., 2018). The significance and implications of the study were discussed to prevent the hemodynamic impairment to patients with HFpEF from the air pollution.

## Methods

### Experimental Measurements

#### Animal Preparation

**Figure 1:**
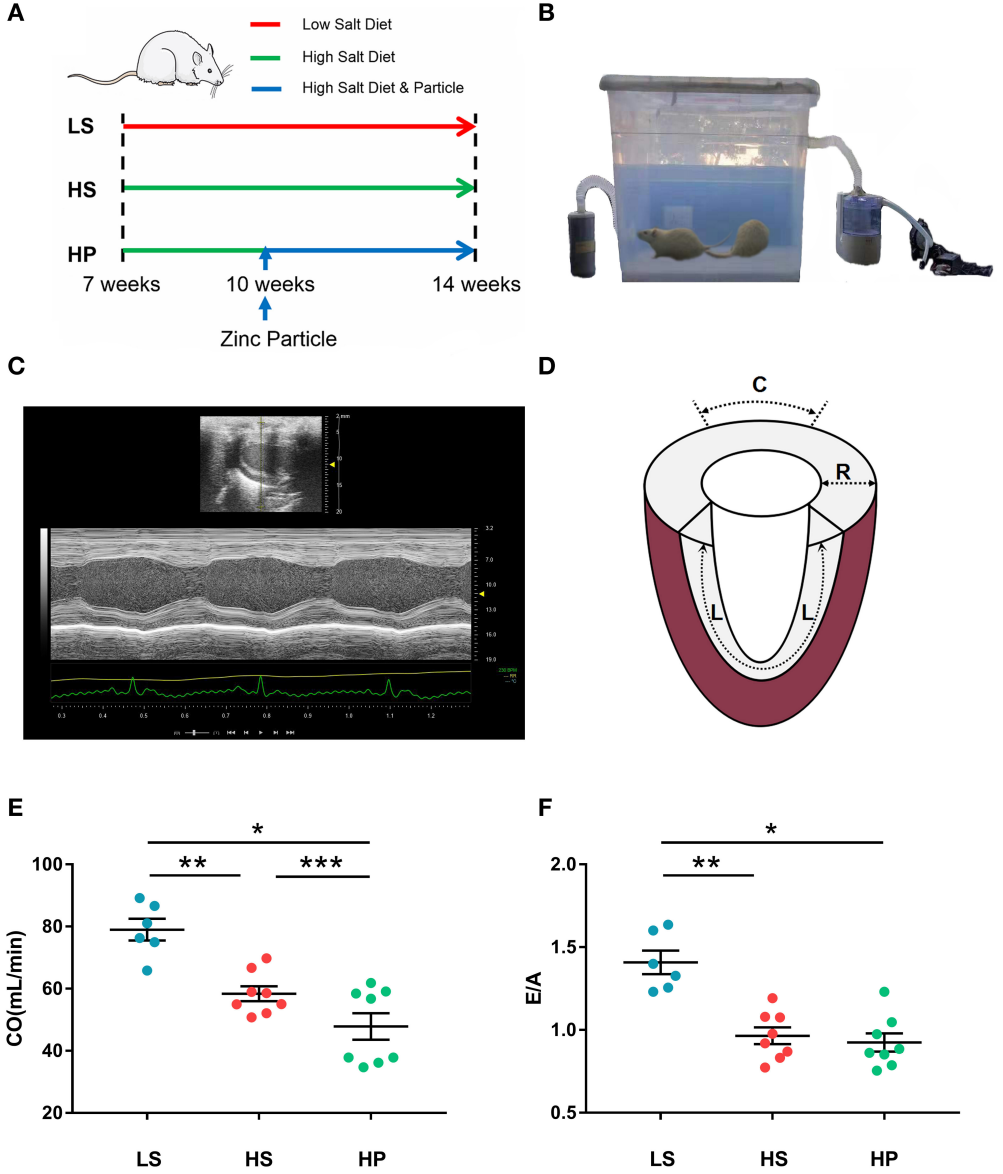
**(A)** Schematic representative of experimental protocol, where 7-week-old DSS rats were fed with low-salt (LS; *n* = 6) or high-salt (HS; *n* = 8) diets for 7 weeks as well as high-salt diet for 7 weeks with inhaling ultrafine zinc particles for 4 weeks (HP; *n* = 8); **(B)** the experimental device for regulating the inhalation of ultrafine zinc particles in the HP group; **(C)** echocardiography in B-mode tracings; **(D)** schematic diagram of strain direction; **(E)** CO and **(F)** E/A in the three groups at the age of 14 weeks, where symbol *means LS vs. HP agree *P* < 0.05, symbol **means LS vs. HS agree *P* < 0.05 and symbol ***means HS vs. HS agree *P* < 0.05.

Experiments were carried out on 7-week-old DSS male rats (Beijing Vital River Laboratory Animal Technology Co., Ltd.). The rat model (24 animals, weighing 300 ± 34 g) was established according to the method in Figure 1A (Klotz et al., 2006; Cho et al., 2017). Two rats died and were not counted. Experimental protocol was consisted of three groups: LS group, HS group, and HP group, where 6 LS animals were fed with low salt diet, i.e., 0.3% sterile NaCl (Beijing KeaoXieli Feed Co., Ltd.) until they were at the age of 14 weeks; 8 HS animals were fed with high salt diet, i.e., 8% sterile NaCl until 14 weeks; and 8 HP animals were also fed with high salt diet until 14 weeks, but started to inhale ultrafine zinc particles (~50 nm, Beijing Deke Daojin Science And Technology Co., Ltd.) with the concentration of about 500 μg/m^3^ (Beckett et al., 2005; Wallenborn et al., 2008) when they were 10-week-old.

An experimental device was designed for regulating the inhalation of ultrafine zinc particles in the HP group, where a sealed chamber was connected with an atomizer of ultrasonic irradiation (YUWELL 402B, Jiangsu Yuyue Medical Equipment & Supply Co., Ltd.) and a carbon filter by two tubes. As shown in Figure 1B, the aerosolized ultrafine zinc particles were mixed with the dry air at a frequency of 1.7 MHz and directed to the chamber. The air flow was monitored by a flow monitor (MF5706, Siargo Co., Ltd.) to regulate the concentration of ultrafine zinc particles. The 10-week-old HP animals inhaled ultrafine zinc particles 3 h per day for 4 weeks. All animals were fed a certain amount of diet and water every day, raised at 26°C indoors, and under 12:12 h light/dark artificial cycle conditions. All animals were raised to 14 weeks of age and underwent the ultrasound measurements. The HP and HS groups showed weakness and decreased activity. All experiments were performed in accordance with Chinese National and Peking University ethical guidelines regarding the use of animals in research, consistent with the NIH guidelines (Guide for the care and use of laboratory animals) on the protection of animals used for scientific purposes. The experimental protocol was approved by the Animal Care and Use Committee of Peking University, China.

#### Echocardiographic Measurements

Rats were anesthetized with ~5% isoflurane and maintained with ~2% isoflurane. Rats were ventilated with room air using a ventilator (Inspira). Echocardiographic measurements of rat hearts, as shown in Figure 1C, were carried out in the three groups (Wu et al., 2017). The images were obtained at 21 MHz using a MS-250 transducer operated by the Vevo2100 Color Doppler Ultrasound Scanner (FUJIFILM Visual Sonics Inc. Canada). Based on M-mode tracings, morphometric parameters, e.g., LVID_d_ and LVID_s_, were measured according to the American Society of Echocardiography leading edge rule (Sahn et al., 1978). These parameters were averaged based on five measurements. Moreover, EF (%) was calculated as: $\frac{\left(LVID_{d}-LVID_{S}\right)}{LVID_{d}}\times 100\%$, in the Vevo LAB image analysis workstation.

#### STE Measurements

In B-mode tracings, 2D grayscale images were obtained from the standard parasternal longitudinal view (Wang et al., 2008). Frame rate is 133 Hz, gain is 20~25 dB, depth is ~20 mm, width is ~23 mm. Three cardiac cycles were recorded. Myocardial deformation measurements were demonstrated using the Vevo LAB image analysis workstation with advanced STE, which tracks natural acoustic markers (called speckles) across the cardiac cycle and estimates velocity vectors. Furthermore, longitudinal and radial strains ($=\frac{L}{L_{0}}$, where *L*_0_ and *L* refer to the baseline length at the R-Wave and the absolute change in length, respectively) and strain rates ($=\frac{V}{L_{0}}$, where *V* is the velocity gradient in the segment) were determined and analyzed by the software across the entire LV over a selected period of cardiac cycles, as shown in Figure 1D.

#### Hemodynamic Measurements

The abdominal aorta (AA), left carotid artery (CA) and femoral artery (FA) were dissected. Perivascular flow probes (Transonic Systems Inc.; relative error of ±2% at full scale) were mounted on FA, CA, and AA to measure the volumetric flow rate. The waves in FA and CA were measured simultaneously to determine the PWV. A 1.4F micromanometer-tipped catheter (Millar Instruments) was inserted through the right carotid artery into the ascending aorta and LV as well as the descending aorta to record pressure waves in 30 cardiac cycles, which was repeated three times. The zero-pressure baseline of the catheter was calibrated in 37°C saline. The catheter and flow probes were monitored with a BIOPAC MP150 (Huo et al., 2018).

#### Histological Evaluation

Nuclear morphology was assessed by Hoechst 33258 dye (Molecular Probes, Germany) as reported in a previous study (Kirshenbaum and de Moissac, 1997). Masson's trichrome and Picro-Sirius Red (PSR) staining were carried out according to standard procedures (Puente et al., 2014; Deng et al., 2016). The density of vascular smooth muscle and collagen fiber content were obtained from the histological staining.

### Mathematic Method

#### Windkessel Analysis

We obtained the time-averaged pressure and flow over a cardiac cycle (P_mean_ and Q_mean_) based on the measurements of pressure and flow waves of carotid artery and abdominal aorta. The cardiac output, CO, equaled to Q_mean_ × 60 s. The arterial tree is modeled as an elastic chamber (Windkessel) with total compliance, C, and peripheral resistance, R (Westerhof et al., 2009). The latter is calculated as:

(1) $$\begin{array}{r} R=\left(P_{ao,mean}-P_{ven,mean}\right)/Q_{mean}\approx P_{ao,mean}/Q_{mean} \end{array}$$

where *P*_*ao, mean*_ and *P*_*ven, mean*_ are the mean aortic and venous pressure, respectively, because of negligible venous pressure. In the diastolic period, the blood pressure decays with a power form (Westerhof et al., 2009):

(2) $$\begin{array}{r} p\left(t\right)=p_{1}e^{-\;\frac{t}{RC}} \end{array}$$

where *p*_1_ is the peak blood pressure at the time *t*_1_. Taking the natural log function, Equation (2) can be written as:

(3) $$\begin{array}{r} \ln p\left(t\right)=-\frac{t}{RC}+\ln\;p_{1} \end{array}$$

Provided the slope of *k* between ln*p*(*t*) and *t*, total compliance, C, is obtained:

(4) $$\begin{array}{r} C=-\frac{1}{R\cdot k} \end{array}$$

On the other hand, PWV is used to evaluate the arterial stiffness. Similar to a previous study (Rogers et al., 2001), *PWV* = *x*/*t*, where *x* is the distance between carotid and femoral artery and *t* is the foot-to-foot delay time between carotid and femoral flow waves. The “foot” is defined as the point where a sharp systolic upstroke begins.

#### Womersley Analysis

Similar to a previous study (Huo et al., 2018), the equation for the pulsatile flow velocity profile across the lumen, *u*(*r, t*), is given as:

(5) $$\begin{array}{r} u\left(r,t\right)=REAL\left[\frac{2Q(0)\left(R^{2}-r^{2}\right)}{\pi R^{4}}+\sum_{\omega \;=\;1}^{\infty }\frac{\frac{Q\left(\omega \right)}{\pi R^{2}}\cdot (1-\frac{J_{0}(\Lambda r/R)}{J_{0}(\Lambda )}}{1-\frac{2J_{1}(\Lambda )}{\Lambda J_{0}(\Lambda )}}e^{i\omega t}\right] \end{array}$$

where *r* is the radial coordinate, *R* is the radius of artery, ∧^2^ = *i*^3^α^2^, $\alpha =R\sqrt{\frac{\omega \rho }{\mu }}$, $q_{measured}\left(t\right)=Q\left(\omega \right)e^{i\omega t}$, ω is the angular frequency after Fourier transformation, *J*_0_ is a Bessel function of zero order and first kind, and *J*_1_ is a Bessel function of first order and first kind. Accordingly, wall shear stress (WSS), τ(*R, t*), and oscillatory shear index (OSI) for pulsatile blood flow can be written as:

(6) $$\begin{array}{r} \tau \left(R,t\right)=REAL\left(\frac{4\mu }{\pi R^{3}}Q\left(0\right)-\sum_{\omega =1}^{\infty }\frac{\frac{\mu Q\left(\omega \right)}{\pi R^{3}}\cdot \frac{{\wedge J}_{1}\left(\wedge \right)}{J_{0}\left(\wedge \right)}}{1-\frac{{2J}_{1}\left(\wedge \right)}{\wedge J_{0}\left(\wedge \right)}}e^{i\omega t}\right) \end{array}$$

(7) $$\begin{array}{r} OSI=\frac{1}{2}\left(1-\frac{\left|\frac{1}{T}\int_{0}^{T}\tau \left(R,t\right)\right|}{\frac{1}{T}\int_{0}^{T}|\tau \left(R,t\right)|}\right) \end{array}$$

The viscosity (μ) and density (ρ) were assumed to be 4.0 cp and 1.06 g/cm^3^, respectively. Moreover, relative residence time (RRT) reflects the residence time of flow particles near the wall and is recommended as a single metric of low oscillating shear stress, which is expressed as follows:

(8) $$\begin{array}{r} RRT=\frac{1}{\left(1-2\cdot OSI\right)\cdot TAWSS} \end{array}$$

### Statistical Analysis

The experimental measurements were repeated three times and averaged per animal. The mean and standard error (mean ± SE) were computed by averaging over all animals in each group. One-Tailed Test was used to compare the various morphometric and hemodynamic parameters between different groups, where *p* < 0.05 represented statistically significant difference.

## Results

Figures 1E,F show the CO and E/A in LS, HS, and HP groups for 7 weeks after the experiment began. Echocardiographic measurements show that HS and HP groups have a significant decrease of CO (78.98 ± 3.48 in LS vs. 58.36 ± 2.39 in HS vs. 47.84 ± 4.28 in HP) and E/A ratio (1.41 ± 0.07 in LS vs. 0.96 ± 0.05 in HS vs. 0.92 ± 0.06 in HP) as compared with the LS group. A decrease in CO means lower LV pumping function. Table 1 lists morphometric and hemodynamic parameters in the LV of the three groups, which shows an increase of LV ESV and LVEDP, but a decrease of EF and SV in HS and HP groups as compared with the LS group. The diastolic dysfunction occurs because of the morphometric and hemodynamic changes in HS and HP groups despite no significant difference between the two groups.

**Table 1:** Morphometric and hemodynamic parameters in the LV of LS, HS, and HP groups.

**Groups**	**LS (*n* = 6)**	**HS (*n* = 8)**	**HP (*n* = 8)**
**MORPHOMETRIC PARAMETERS**			
LVIDs (mm)	2.99 ± 0.34^*^	3.86 ± 0.20^**^	3.92 ± 0.15
LVID_d_ (mm)	6.88 ± 0.13	6.89 ± 0.16	6.91 ± 0.10
ESV (μL)	39 ± 9^*^	66 ± 6^**^	68 ± 6
EDV (μL)	246 ± 11	252 ± 11	255 ± 6
SV (μL)	207 ± 8^*^	186 ± 5^**^	173 ± 10
EF (%)	84.7 ± 3.5^*^	72.7 ± 2.4^**^	72.9 ± 2.2
**HEMODYNAMIC PARAMETERS**			
LVSP (mmHg)	133 ± 12^*^	161 ± 4^**^	170 ± 7
LVEDP (mmHg)	3.5 ± 1.4^*^	7.5 ± 1.3^**^	7.9 ± 1.2

* Means LS vs. HP agree P < 0.05,

** *means LS vs. HS agree P < 0.05*.

**Figure 2:**
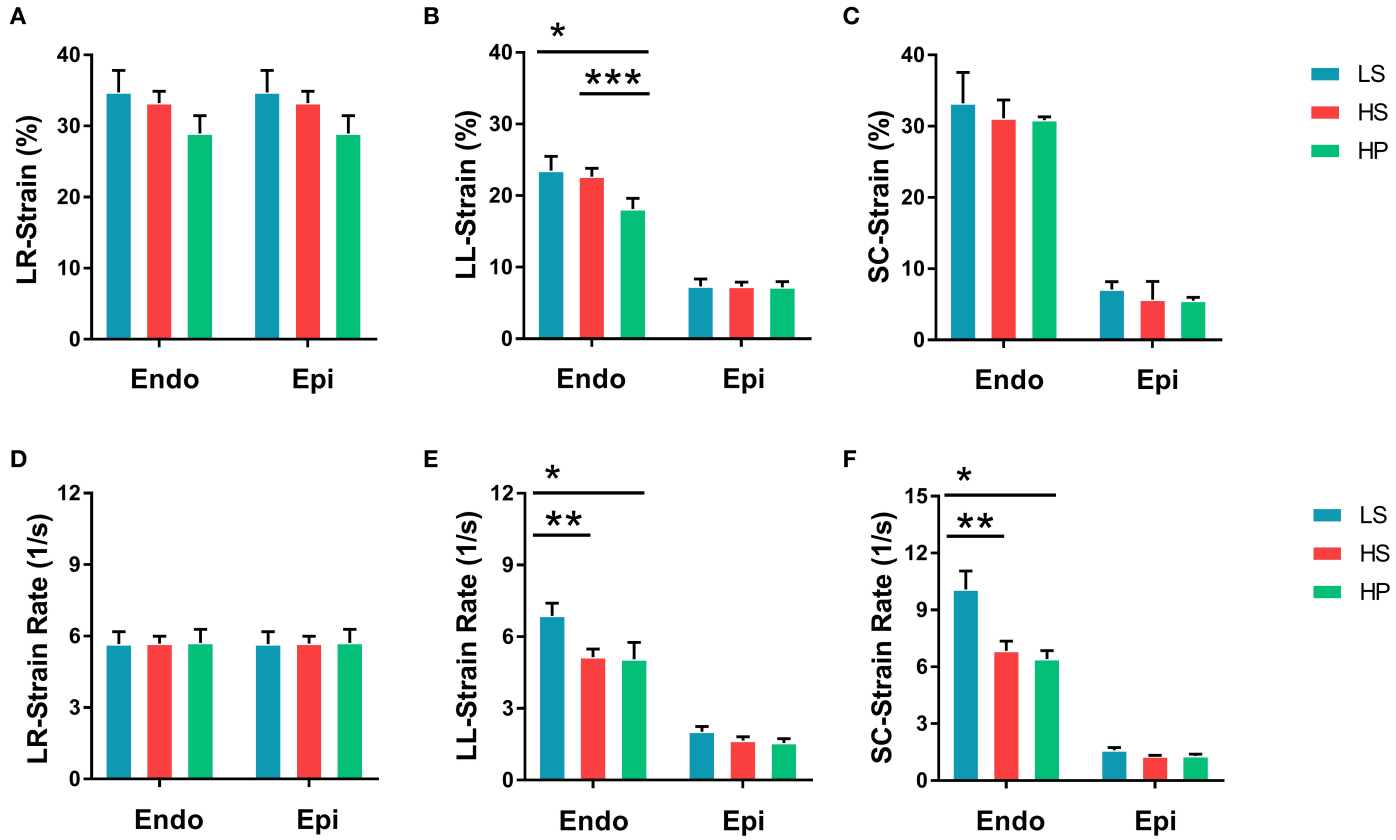
**(A)** Peak radial strains, **(B)** peak longitudinal strains, and **(C)** peak circumferential strains on endocardium and epicardium of LS, HS, and HP rat hearts at the age of 14 weeks; and **(D)** peak radial strain rates, **(E)** peak longitudinal strain rates, and **(F)** peak circumferential strain rates corresponding to **(A–C)**. *Means LS vs. HP agree *P* < 0.05, **means LS vs. HS agree *P* < 0.05, ***means LS vs. HS agree *P* < 0.05.

Figures 2A–C show peak strains and Figures 2D–F show peak strain rates in radial, longitudinal, and circumferential directions on endocardium and epicardium of the three groups. There are significantly higher strains and strain rates in longitudinal and circumferential directions on the endocardium than the epicardium. The strain on endocardium or epicardium decreases in a sequence of LS, HS, and HP rats. HS and HP rats have lower strain rates in longitudinal and circumferential directions on the endocardium than the LS group.

**Figure 3:**
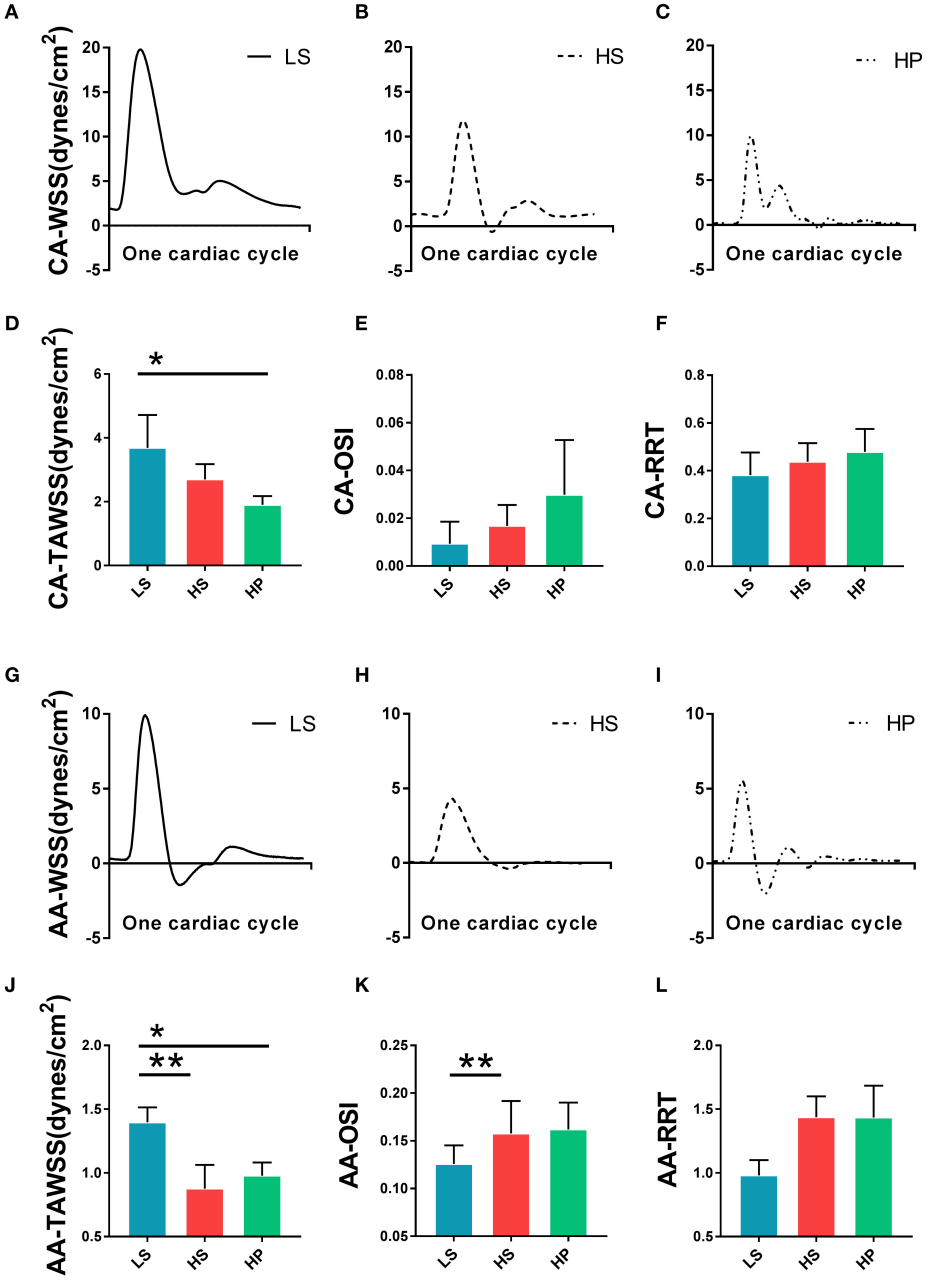
Transient WSS in the carotid artery of **(A)** LS, **(B)** HS, and **(C)** HP groups; **(D)** TAWSS, **(E)** OSI, and **(F)** RRT in the carotid artery of the three groups; Transient WSS in the abdominal aorta of **(G)** LS, **(H)** HS, and **(I)** HP groups; and **(J)** TAWSS, **(K)** OSI, and **(L)** RRT in the abdominal aorta of the three groups. *Means LS vs. HP agree *P* < 0.05, **means LS vs. HS agree *P* < 0.05.

**Figure 4:**
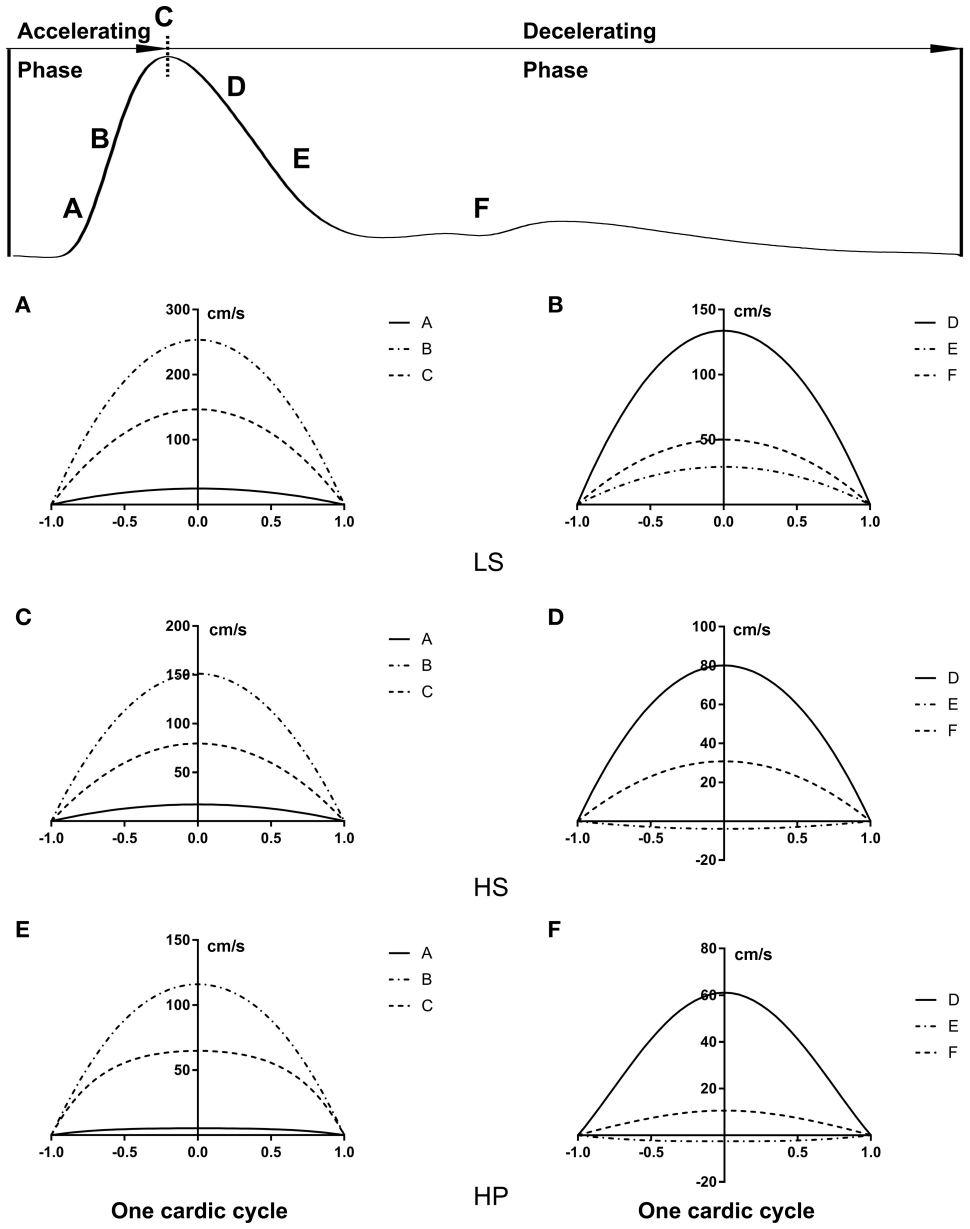
Flow velocity profiles (cm/s) in the carotid artery at various time instances during **(A,C,E)** accelerating and **(B,D,F)** decelerating periods in **(A,B)** LS, **(C,D)** HS, and **(E,F)** HP groups.

**Figure 5:**
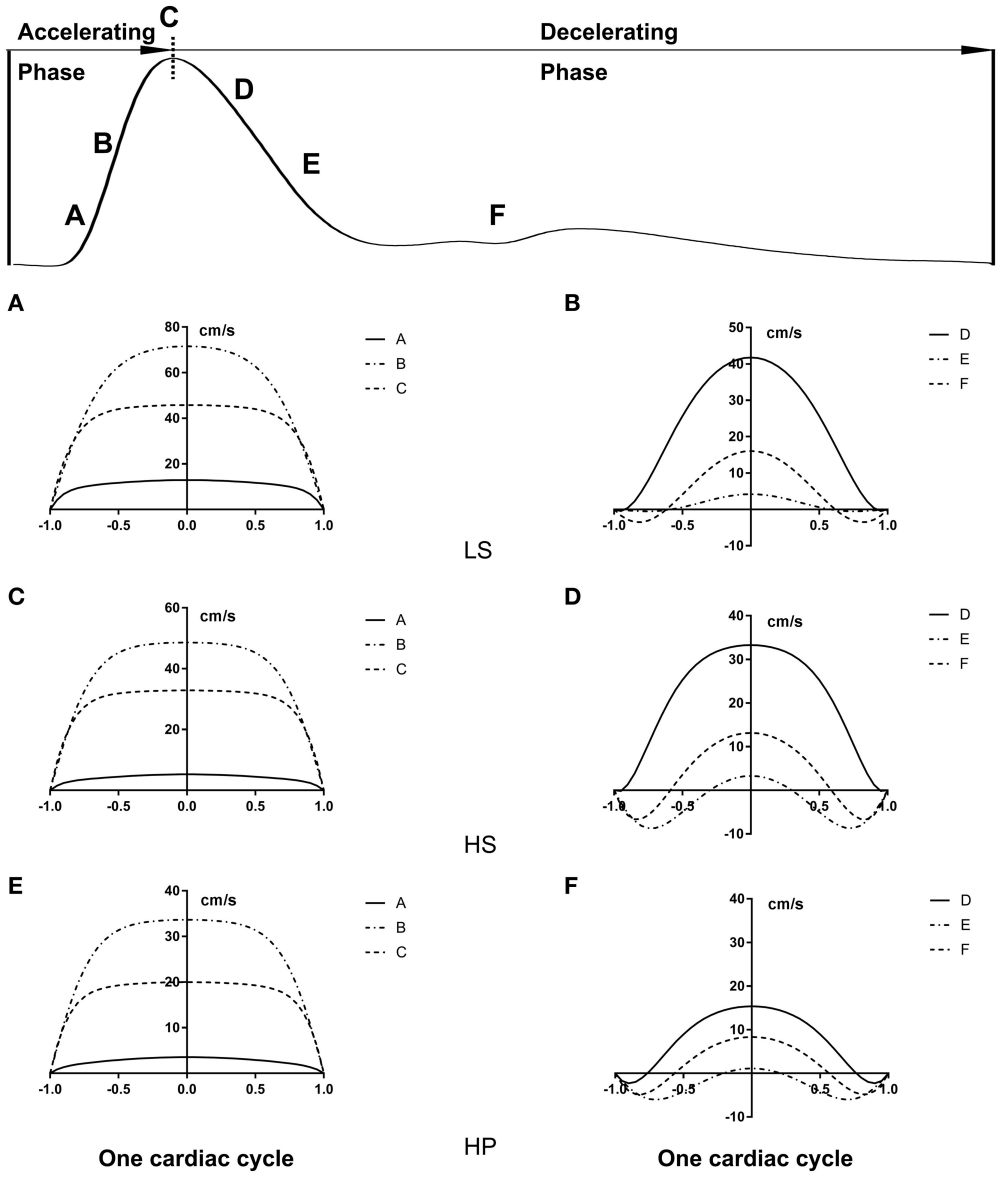
Flow velocity profiles (cm/s) in the abdominal aorta at various time instances corresponding to Figure 4.

Figures 3A–C show transient WSS in the carotid artery of representative LS, HS, and HP rats, respectively. Figures 3D–F show TAWSS, OSI, and RRT in LS, HS, and HP groups. Accordingly, Figures 3G–L show these hemodynamic parameters in the abdominal aorta. In the carotid artery, TAWSS decreases, and OSI and RRT increases in a sequence of LS, HS, and HP rats. In the abdominal aorta, HS and HP rats have lower TAWSS and higher OSI and RRT than the LS despite no statistical difference between HS and HP groups. Figures 4, 5 show flow velocity profiles in the carotid artery and abdominal aorta during the accelerating and decelerating periods in representative LS, HS, and HP rats. The flow reversal occurs in the decelerating period.

**Figure 6:**
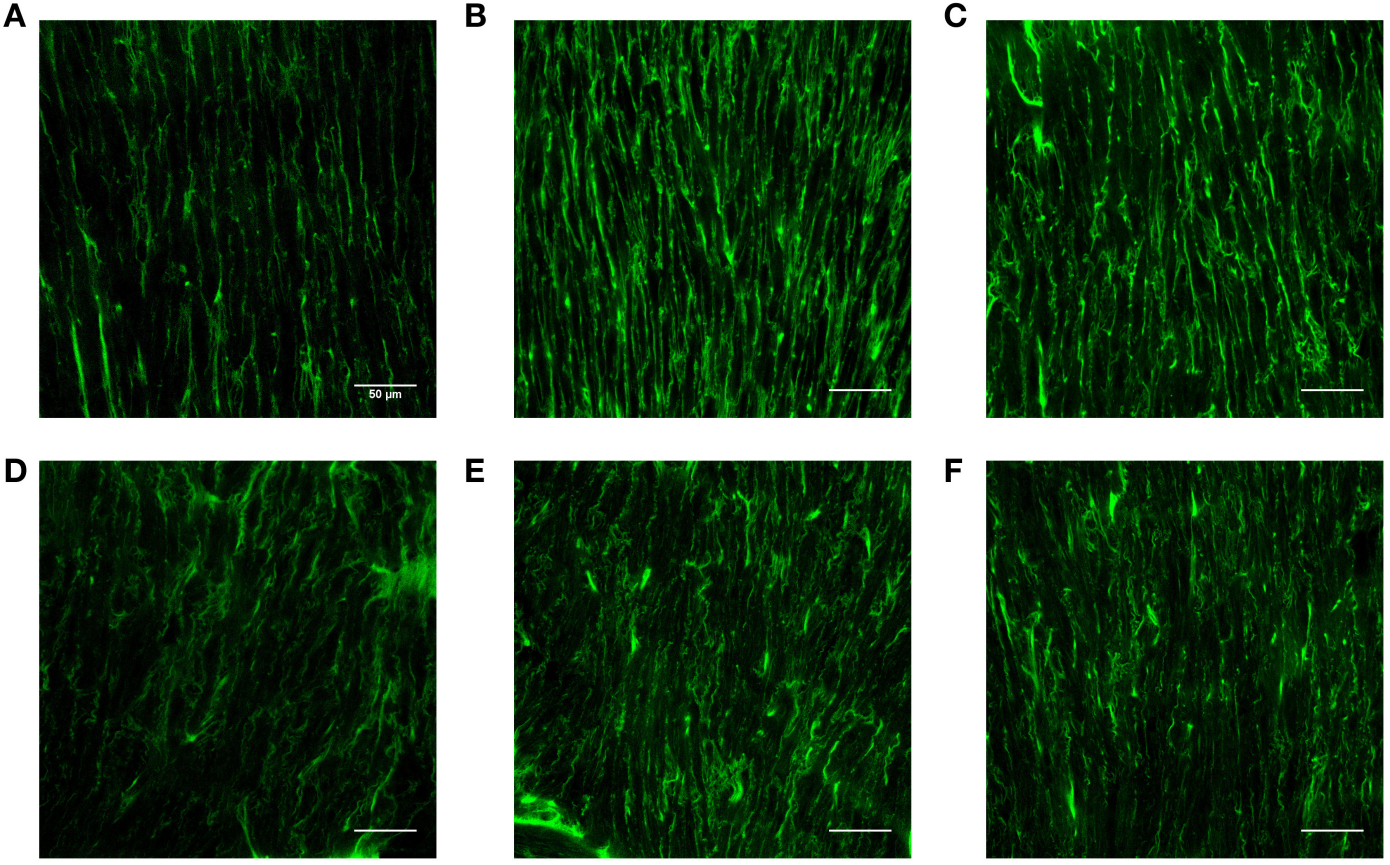
Collagen fiber distribution in the carotid artery of **(A)** LS, **(B)** HS, and **(C)** HP groups and in the abdominal aorta of **(D)** LS, **(E)** HS, and **(F)** HP groups.

Table 2 lists hemodynamic parameters in peripheral arteries of the three groups. The systolic and diastolic aortic pressures and PWV increase while the blood flow rate decreases in a sequence of LS, HS, and HP rats. In the carotid artery and abdominal aorta, the systemic resistance increases and the total vascular compliance decreases in a similar sequence. On the other hand, Figure 6 shows the collagen fiber distribution in the carotid artery and abdominal aorta of representative LS, HS, and HP rats. Table 3 lists the corresponding VSMC density and collagen fiber content ratio. The decreased VSMC density and the increased collagen fiber ratio can reasonably explain vascular dysfunctions and hemodynamic impairments in HS and HP rats as compared with the LS group.

**Table 2:** Hemodynamic parameters in the carotid artery and abdominal aorta.

**Groups**	**Items**	**LS**	**HS**	**HP**
	ESP (mmHg)	129 ± 5^*^	161 ± 8^**^	172 ± 6
	EDP (mmHg)	83 ± 6^*^	109 ± 6^**^	122 ± 8
	PWV (m/s)	7.9 ± 3.9^*^	14.4 ± 13.5^**^	15.9 ± 15.4
CA (*n* = 6)	Flow rate (mL/s)	0.28 ± 0.09^*^	0.21 ± 0.02	0.12 ± 0.01^***^
	Diameter (mm)	0.94 ± 0.04	1.03 ± 0.02	1.09 ± 0.05
	R (mmHg·s/mL)	591 ± 112^*^	704 ± 69	1,036 ± 110^***^
	C (10^−3^mL/mmHg)	0.77 ± 0.54^*^	0.58 ± 0.26	0.34 ± 0.24^***^
AA (*n* = 3)	Flow rate (mL/s)	0.47 ± 0.03^*^	0.35 ± 0.02^**^	0.32 ± 0.05
	Diameter (mm)	2.03 ± 0.04	2.14 ± 0.09	2.14 ± 0.002
	R (mmHg·s/mL)	178 ± 16^*^	339 ± 66^**^	430 ± 70
	C (10^−3^mL/mmHg)	2.30 ± 0.21	1.90 ± 0.31	1.73 ± 0.34

* Means LS vs. HP agree P < 0.05,

** means LS vs. HS agree P < 0.05,

*** *means HS vs. HP agree P < 0.05*.

**Table 3:** Mean VSMC density and collagen fiber content ratio in the carotid artery and abdominal aorta.

**Position**	**Groups**	**CA**	**AA**
Mean VSMC density	LS	1.60 ± 0.48^*^	1.09 ± 0.08^*^
(number/1,000 μm^2^)	HS	1.16 ± 0.05^**^	0.84 ± 0.12^**^
	HP	1.13 ± 0.02	0.82 ± 0.07
Fiber content ratio	LS	0.16 ± 0.06^*^	0.22 ± 0.04^*^
$\left(\frac{Volume\;of\;collagen\;fibers}{Volume\;of\;vessel\;wall}\right)$	HS	0.36 ± 0.08^**^	0.31 ± 0.10^**^
	HP	0.40 ± 0.06	0.38 ± 0.02

* Means LS vs. HP agree P < 0.05,

** *means LS vs. HS agree P < 0.05*.

## Discussion

This study investigated the effect of inhaling ultrafine zinc particles on LV function and peripheral vascular hemodynamics in rats with HFpEF (the HS group) experimentally and theoretically. The study focused on LV dysfunctions and deteriorated hemodynamic environment in peripheral arteries in the HS and HP groups.

### LV Dysfunctions

The significant elevation of LVSP and LVEDP led to structural changes in the LV of HS and HP groups. LVIDs and ESV were increased and SV, CO, EF, and E/A were reduced in the two groups as compared with the LS group. There was no statistical difference between HS and HP groups albeit HP had more deteriorated structure and function of the LV. These changes supported that HS and HP rats developed multiple pathological features of HFpEF similar to those in patients of hypertension-induced HFpEF (Borlaug and Paulus, 2011).

Exposure to ultrafine particles can lead to dysfunctions of the cardiac autonomic nervous system and systemic inflammation (Rivero et al., 2005; Chuang et al., 2007; Jia et al., 2012). Ultrafine particles can also enter the blood circulation directly and cause systolic and diastolic dysfunctions of the heart (Houston, 2007). Excessive intake of Zinc, a transition metal element, can damage mitochondria and sarcoplasmic reticulum through direct toxic effects, resulting in impaired energy supply of myocytes. This exacerbates myocardial ischemia and hypertrophy in HS-feeding rats and constricts coronary arteries which in turn aggravate myocardial ischemia and hypoxia, further causing myocardial damage and affecting hemodynamics (Schnee and Hsueh, 2000).

Since systolic function is compensatory, the overall cardiac function of the heart remains normal, but the local myocardium may have been impaired in HS and HP rats (Poulsen et al., 2003). Strain and strain rate quantitatively characterize the local myocardial deformation (Jamal et al., 2001). HS and HP rats showed lower strain in radial, longitudinal, and circumferential directions on endocardium and epicardium than the LS group. The inhalation of ultrafine zinc particles further reduced the strain in the longitudinal direction on the endocardium of rats with HFpEF, but had relatively small effects on the epicardium. These findings support the impaired local myocardium in HFpEF, which is worsened by the inhalation of ultrafine zinc particles.

### Hemodynamics in Peripheral Arteries

We investigated the changes of hemodynamic parameters in the abdominal aorta and carotid artery caused by HFpEF, and studied the changes of these parameters after inhalation of ultrafine zinc particles. The Womersley analysis showed that TAWSS in the HS group was ~27% lower than that in LS group while the value in the HP group was ~30% lower than that in the HS group in the carotid artery. Moreover, OSI and RRT in the HS group were ~81 and ~15% higher than that of the LS group while the values in the HP group were ~78 and ~10% higher than the HS group. The inhalation of ultrafine zinc particles significantly worsened the hemodynamic environment in the carotid artery, such as the reduction of WSS, the increase of OSI, and the prolongation of RRT. For the abdominal aorta, TAWSS and OSI/RRT in the LS group was significantly higher and lower than those in HS and HP group despite no significant difference between the two groups. This indicated negligible effects of inhaling ultrafine zinc particles on the aortic hemodynamics. The vessel of small size is predisposed to remodeling as compared with the vessel of large size (Huo et al., 2007; Kodavanti et al., 2008; Huo and Kassab, 2012), which can reasonably explain different hemodynamic changes between the carotid artery and abdominal aorta in rats of HFpEF after the inhalation of ultrafine zinc particles. There are three mechanisms for the damage of ultrafine particles to the cardiovascular system: triggering oxidative stress and inflammation, disrupting the autonomic nervous system, and directly entering the blood circulation (Brook et al., 2010; Cutrufello et al., 2011). These led to the changes of hemodynamic parameters in the abdominal aorta and carotid artery of the HP group as compared with the HS group. These abnormal parameters can result in the endothelial dysfunction, monocyte deposition, microemboli formation, SMC proliferation, and so on (Chiu and Chien, 2011; Huang et al., 2016, 2018; Fan et al., 2019). Hence, excessive intake of ultrafine zinc particles deteriorated the hemodynamic environment in rats of HFpEF.

The ESP and EDP in the HS group increased by about 25 and 32% than the LS group. The pressure in the HP group increased by ~12% as compared with the HS group. The flow rate in the carotid artery and abdominal aorta decreased in a sequence of LS, HS, and HP rats. Based on the Windkessel model, the increase of systemic resistance and the decrease of total vascular compliance as well as the increased PWV characterized the arterial and arteriolar stiffening in rats with HFpEF (i.e., the HS group), which agreed with previous studies (Gandhi et al., 2001; Hundley et al., 2001). Furthermore, the inhalation of ultrafine zinc particles deteriorated the pressure-flow relationship in peripheral arteries and induced more severe vascular stiffening in rats with HFpEF. Combined ventricular-arterial stiffening resulted in afterload elevation, which could then feedback to further impair LV diastolic functions. Hence, the inhalation of ultrafine zinc particles accelerated the development of HFpEF.

On the other hand, the Womersley analysis showed velocity profiles in the carotid artery and abdominal aorta at various time instances over a cardiac cycle. There were parabolic and blunt profiles in the carotid artery and abdominal aorta of the three groups, respectively, during the accelerating period. The velocity profile changed significantly during the decelerating period and resulted in different patterns among the three groups. The peak flow velocity decreased and the flow reversal became stronger in the carotid artery and abdominal aorta in a sequence of LS, HS, and HP rats. Increased frequency of refluxes (HP group with the highest OSI) was associated with a sustained activation of a number of atherogenic genes in vascular endothelial cells (Chiu and Chien, 2011). This supported the deteriorated hemodynamic environment in rats of HFpEF caused by the inhalation of ultrafine zinc particles.

### Histological Analysis in Vessel Wall

The pulsatile nature of blood pressure and flow creates hemodynamic stimuli in the forms of cyclic stretch and shear stress, exerting continuous influences on constituents of the blood vessel wall (Nichols and McDonald, 2011). The morphology of SMC is the most rounded in the HP group, followed by the HS group. The cell density per unit area decreased in a sequence of LS, HP, and HS rats. The increased content of collagen fiber indicated the fibrosis of the vascular wall in the HS group, which was worsened in the HP group. The low WSS and high OSI stimulated various intracellular and intercellular signals to regulate SMC functions such as migration, remodeling (Haga et al., 2007) and rearrange the microstructure (Hariton et al., 2007). This reasonably explained the increase of PWV and the decrease of total vascular compliance in the HP group.

### Critique of Study

We used the ultrafine zinc particles and assumed a constant concentration, which was different from the measured PM0.1 composition. The arterial distensibility needed to be incorporated into the cardiovascular system for a more systematic analysis. A three-dimensional computational fluid dynamic model, coupled with morphometric data of the peripheral arteries, should be used to investigate the high blood pressure and the effects of inhaling various metal particles on the hemodynamics in the following studies.

## Conclusions

Excessive intake of ultrafine zinc particles resulted in the increase of arterial pressure, PWV and impedance while the decrease of total vessel compliance in peripheral arteries of rats with HFpEF. It also led to the local myocardium dysfunctions in HFpEF. The ventricle-vascular uncoupling due to the inhalation of ultrafine zinc particles impaired the cardiac and cardiovascular functions in HFpEF. This shed light on understanding the hemodynamic mechanisms for potential deterioration of HFpEF due to air pollution.

## Data Availability Statement

All datasets generated for this study are included in the article/supplementary material.

## Ethics Statement

This animal study was reviewed and approved by Animal Care and Use Committee of Peking University.

## Author Contributions

FB and XW contributed equally to this study. LL, PN, WS, and WZ performed experiments. FB, XW, and YC performed the theoretical analysis. FB, XW, and YH drafted the manuscript. YH and WT reviewed the manuscript. All authors approved it for publication.

### Conflict of Interest

The authors declare that the research was conducted in the absence of any commercial or financial relationships that could be construed as a potential conflict of interest.

## Abbreviations

HFpEF: heart failure with preserved ejection fraction
HF: heart failure
DSS: dahl salt-sensitive
HS: high-salt
LS: Low-Salt
CO: cardiac output
LV: left ventricle
SV: stroke volume
EF: ejection fraction
ESV: end-systolic volume
EDV: end-diastolic volume
LVID_s_: LV internal diameter in systole
LVID_d_: LV internal diameter in diastole
CA: carotid artery
AA: abdominal aorta
PWV: pulse wave velocity
VSMC: vascular smooth muscle cell
WSS: wall shear stress
TAWSS: time-average wall shear stress
OSI: oscillating shear index
RRT: relative residence time.
